# What Can Brinley Plots Tell Us About Cognitive Aging? Exploring Simulated Data and Modified Brinley Plots

**DOI:** 10.3389/fpsyg.2022.797583

**Published:** 2022-02-18

**Authors:** Jessica Nicosia, Emily R. Cohen-Shikora, Michael J. Strube

**Affiliations:** ^1^Knight Alzheimer’s Disease Research Center, Washington University, St. Louis, MO, United States; ^2^Department of Psychological and Brain Sciences, Washington University, St. Louis, MO, United States

**Keywords:** cognitive aging, Brinley plots, attention, Stroop, multi-level model

## Abstract

Cognitive aging researchers have been challenged with demonstrating age-related effects above and beyond global slowing ever since Cerella raised this issue in 1990. As the literature has made clear, this has indeed proved to be a difficult task and continues to plague the field. One way that researchers have attempted to test for disproportionate age differences across task conditions is by using Brinley plots, or plotting the mean response latencies of older adults against the mean latencies for younger adults. The simplicity and large proportion of variance accounted for by these models has led to the widespread use of Brinley plots over the years. However, as systematically tested here through eight cases of simulated data, it is clear that the Brinley technique is not well suited to either identify or display the underlying structure of datasets examining age-related differences in attentional control. Some of the problems with conventional Brinley plots can be resolved by using a modified Brinley plot that includes study-specific slopes linking trial types and a no-age-difference reference line. Multilevel models find all of the relevant effects, especially if applied to trial-level data, and have the advantage of incorporating study-level moderators that might account for slope heterogeneity. Ultimately, we encourage fellow cognitive aging researchers to access the code and data for this project on OSF (https://osf.io/zxus8/) and employ the use of multilevel models over Brinley plots.

## Introduction

Attentional control is a critical component of everyday behavior that allows one to focus on relevant information and resist distracting stimuli in the environment. In the context of laboratory studies, attentional control is often assessed by examining response times and comparing performance across control and interference conditions. One task commonly used to assess attentional control, and used throughout this paper to illustrate the issue at hand, is the Stroop color-naming task (Stroop, 1935), in which participants must resist the well-practiced act of *reading* a word in favor of responding to the *color* of that word. The reaction time and/or accuracy difference between incongruent (interference) trials (in which the word and color do not match, for example, the word “red” displayed in blue) and congruent (baseline) trials (in which the word and color match, for example, the word “red” displayed in red) is known as the *Stroop Effect*, and is examined in group and individual differences research as a metric of attentional control (see MacLeod, 1991). Larger Stroop effects relative to a comparison group are taken as evidence for an attentional control deficit, for example in individuals with Schizophrenia (Cohen and Servan-Schreiber, 1992; Perlstein et al., 1998; Barch et al., 1999) or attention deficit hyperactivity disorder (ADHD; Homack and Riccio, 2004; Van Mourik et al., 2005) and, most relevant to the current study, older adults (e.g., Comalli et al., 1962; Cohn et al., 1984; Panek et al., 1984; Hartley, 1992; Spieler et al., 1996).

However, directly comparing the Stroop effect, or other attentional control task interference effects, across different populations can be challenging given that differences between conditions increase as a function of overall speed (Faust et al., 1999). In other words, larger interference effects for one group as compared to another may reflect differences in processing speed rather than any mechanistic change in attentional control (e.g., Salthouse, 1996). Therefore, it is critical to control or account for differences in overall speed between groups of interest. One way that researchers have done this is by using Brinley plots (Brinley, 1965) to assess age differences in cognitive performance above and beyond general slowing (Cerella, 1985, 1990; Hale et al., 1987, 1991; Myerson et al., 1990). Brinley plots display younger and older (or faster and slower) participants’ mean reaction times against each other for each task or condition, to determine whether one or two regression lines fit the data. If a single linear component explains a majority of the variance, the implication is that a general slowing parameter explains most of the age-related variance and specific age-related mechanisms could not account for additional variance above and beyond that. These plots have shown some of the strongest correlations in all of psychology. For example, Hale et al. (1991) reported a model in which the regression line successfully accounted for 95.6% of the variance, concluding that general slowing was driving nearly all age-related differences in their tasks.

In the related *meta-analytic* Brinley techniques, researchers assess the role of general slowing across many studies by plotting mean *study-level* response latencies for each condition as a function of age (e.g., Verhaeghen and De Meersman, 1998; Verhaeghen, 2011; Rey-Mermet and Gade, 2018). Comparison of models with and without the interaction term present is then used to test whether one or two lines are necessary to account for the relationship between younger and older adults. Like the use of Brinley plots described above, if one line is sufficient, then the apparent age differences in the interaction effect of interest are attributable mainly to processing speed differences, whereas if separate lines are necessary for younger and older adults, then this substantiates the claim that there may be differences between the groups above and beyond general slowing.

**FIGURE 1:**
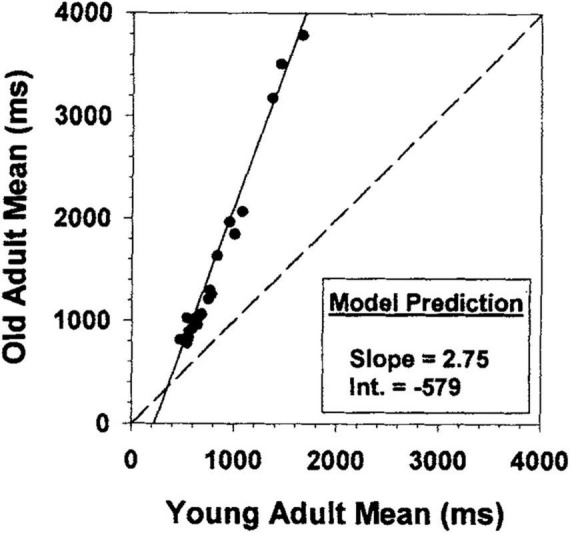
Brinley plot from Faust et al. (1999). Brinley plot from Faust et al. (1999) with data from 26 different studies drawn from Hale et al. (1995).

Despite the widespread use of Brinley plots, and the large correlations produced by such models, there is strong evidence that researchers should use caution when interpreting their results in the context of assessing group differences above and beyond general slowing, particularly with Stroop data. Indeed, this discrepancy in researchers’ interpretations of raw data vs. Brinley plot models has led to disagreement regarding the magnitude of interference effects in healthy older adults compared to younger adults, and thus controversy surrounding conclusions regarding deficits (or the lack thereof) in attentional control. Researchers have used Brinley plot meta-analyses on data from various attentional control tasks and found that, for the majority of tasks, one regression line sufficiently captured the relationship between younger and older adult data (Verhaeghen and De Meersman, 1998; Verhaeghen, 2011; Rey-Mermet and Gade, 2018; see Figure 1 for an example). They therefore concluded that age differences in interference may be an artifact of age-related differences in general processing speed.

One might argue that Brinley plots and general slowing are no longer a relevant issue for cognitive aging researchers. However, it is important to note that papers arguing that age differences in selective attention, amongst other aspects of cognition, do not exist above and beyond the effects of general slowing are still being published and cited. For example, Rey-Mermet and Gade (2018) recently published a manuscript again calling into question older adults’ inhibitory deficit on the basis of Brinley plot analyses which has already been cited well over 100 times. Thus, although the question of how best to examine age differences in cognition accounting for general slowing has existed since the naissance of the Brinley plot in 1965 and Cerella’s publications on the matter in mid 80s to late 90s, it is clear that this issue persists today and requires further understanding and resolution.

To this point, Nicosia et al. (2021) recently tested the veracity of this conclusion by conducting a meta-analysis using trial-level data from 2,896 participants across 33 different computerized, color-naming Stroop task studies with multiple dependent variables (including, most importantly, standardized response times^1^). We conducted meta-regression analyses on a wide set of dependent measures that control for general slowing, tested for publication bias, and examined four potential methodological moderators (publication status, location of data collection, proportion congruence bias, and neutral trial type). We also conducted linear mixed-effect modeling allowing the intercept to vary randomly for each participant, thereby accounting for individual differences in processing speed and directly compared the results of these models to Brinley plot analyses. We found that all analyses except for the Brinley (and related State-Trace) techniques indicated evidence of a disproportionate Stroop effect in older adults, compared to younger adults. Although the results of our meta-analysis were compelling in demonstrating a larger Stroop effect for older adults, the question remains as to *why* Brinley-based techniques lead researchers to the conclusion that Stroop effects in younger and older adults are simply linear functions of each other, whereas all other techniques provide evidence indicating disproportionate age differences in the Stroop effect (even after controlling for general slowing in a host of different ways, see Nicosia et al., 2021 for more information).

One study which attempted to explore this issue did so through the use of simulated data. Perfect (1994) generated data to test the efficacy of Brinley plots and showed that their apparent success in accounting for large proportions of variance is misleading such that (a) Brinley plots fail to detect interactions when data are specifically generated not to conform to a single function and (b) the parameter values obtained differ significantly from the underlying functions used to create the data. Thus, it has been suggested that the large amount of variance captured by a single regression line (vs. two) is not itself sufficient to support the strong claims regarding global models of cognitive aging (see Fisk et al., 1992; Cerella, 1994; Fisk and Fisher, 1994; Myerson et al., 1994; Perfect, 1994, for more on this debate).

Nevertheless, there remains a dearth of research investigating the different conditions under which Brinley plots show disproportionate group differences and converge with other analysis techniques. Thus, the present study explores this issue using simulated Stroop task data and provides researchers with simple modifications which can be made to the Brinley plot to make study-level effects more apparent.

## The Current Study

The current study aimed to extend upon the work of Perfect (1994) and Nicosia et al. (2021) by examining the circumstances necessary to produce converging results indicating disproportionate group effects (i.e., an Age by Trial Type interaction) using Brinley analyses and multilevel models. Specifically, the current study generates simulated Stroop data with different characteristics systematically selected to explore the implications of Brinley plots in contrast to multilevel models for the same data where the underlying structure is specified. Two questions are addressed. First, when it is known that Age by Trial Type effects are present in the data, why don’t Brinley plots show them? Second, under what conditions *will* Brinley plots show a significant Age by Trial Type interaction?

As mentioned by Perfect (1994), a major benefit of conducting studies using simulated data is that it allows for researchers to establish, in advance, the nature of the underlying relationships in the data. Specifically, for any given set of relationships, data can be generated and then used to compare the results of different analytical approaches. Therefore, several “cases” of simulated data are presented (see Table 1). The data for all cases were generated so that the group and condition means have the expected pattern and that the assumed intercorrelations are present.

**TABLE 1:** Simulated data parameters.

Data	Brief description	Young congruent/incongruent	Old congruent/incongruent	N/Group	# Trials	# Studies
Case A	All studies had same interaction	700 (150)/800 (150)	750 (200)/900 (200)	50	20	50
Case B	Increased interaction magnitude	700 (150)/800 (150)	750 (200)/1,000 (200)	50	20	50
Case C	No interaction	700 (150)/800 (150)	750 (200)/850 (200)	50	20	50
Case D	Only main effect of trial type	700 (150)/900 (150)	700 (150)/900 (150)	50	20	50
Case E	Only main effect of age	700 (150)/700 (150)	900 (150)/900 (150)	50	20	50
Case F	All null effects	800 (150)/800 (150)	800 (150)/800 (150)	50	20	50
Case G	Random study-level interaction sizes	700 (150)/800 (150)	750 (200)/900 (200)	50	20	50
Case H	Stronger interactions for some studies	700 (150)/800 (150)	750 (200)/900 (200)	50	20	50

*Mean (SD).*

For each case of simulated data, we directly compare the results across analysis techniques given the same input data with known underlying features. First, we examined Brinley analyses and the standard Brinley plot. Next, we plotted the data using a modified Brinley plot to better display the discrepancies in results across models. The modified Brinley plot was created to overcome the pitfalls of the standard Brinley plot in displaying the nature of study-level effects.

**FIGURE 2:**
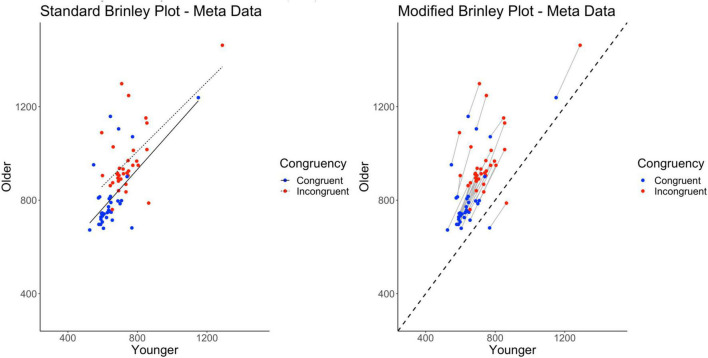
Standard and modified Brinley plots—Nicosia et al. (2021) data. Standard and modified Brinley plots (left and right, respectively) for the meta data from Nicosia et al. (2021).

To demonstrate the necessity of the modified Brinley plot, introduced here, we use the data from Nicosia et al. (2021) to illustrate several specific problems with the standard Brinley plot (specifically, in the context of meta-analysis data examining age differences in the Stroop effect). The left side of Figure 2 shows the standard Brinley plot for the Nicosia et al. (2021) data. Replicating the typical pattern seen in prior work (Verhaeghen and De Meersman, 1998; Verhaeghen, 2011; Rey-Mermet and Gade, 2018), the standard Brinley plot indicates that a single regression line captures the majority of the variance (see left side of Table 2). However, as Nicosia et al. (2021) describe, this sharply contradicts the findings of the multilevel model which indicated clear support for disproportionate age differences in the Stroop effect (see right side of Table 2).

**TABLE 2:** Model comparison and trial-level multilevel model results.

Data	Model	Restricted vs. Full model comparison						Trial-level multilevel models				
		df	*R* ^2^	AIC	BIC	deviance	Δχ^2^	Predictor	Estimate	Standard error	*t*	*P*
Nicosia et al. (2021)	Restricted	5	0.98	727.81	738.76	717.81		Age	18.68	3.73	5.01	**<0.001**
Meta data	Full	6	0.98	729.57	742.7	717.57	0.25	Trial Type	87.04	0.39	221.87	**<0.001**
								Age * Trial type	53.23	0.69	76.79	**<0.001**
Case A	Restricted	5	0.99	757.65	770.68	747.65		Age	47.4	1.87	25.33	**<0.001**
	Full	6	0.99	758.35	773.98	746.35	1.31	Trial type	99.84	1.31	76.25	**<0.001**
								Age * Trial type	48.23	2.2	21.95	**<0.001**
Case B	Restricted	5	0.99	747.80	760.83	737.80		Age	47.40	1.87	25.33	**<0.001**
	Full	6	0.99	749.52	765.15	737.52	0.28	Trial type	98.84	1.31	75.18	**<0.001**
								Age * Trial type	151.16	2.2	68.79	**<0.001**
Case C	Restricted	5	0.99	760.30	773.33	750.30		Age	49.85	1.87	26.7	**<0.001**
	Full	6	0.99	762.11	777.74	750.11	0.18	Trial type	98.76	1.31	75.44	**<0.001**
								Age * Trial type	0.10	2.19	0.04	0.96
Case D	Restricted	5	0.99	743.20	756.22	733.20		Age	1.57	1.6	0.98	0.33
	Full	6	0.99	744.69	760.33	732.69	0.50	Trial type	200.88	1.32	152.63	**<0.001**
								Age * Trial type	0.15	1.86	0.08	0.94
Case E	Restricted	5	0.99	727.17	740.19	717.17		Age	200.72	1.61	124.55	**<0.001**
	Full	6	0.99	727.93	743.56	715.93	1.23	Trial type	0.42	1.32	0.32	0.75
								Age * Trial type	−1.95	1.86	−1.05	0.30
Case F	Restricted	5	0.99	726.97	739.99	716.97		Age	0.34	1.58	0.21	0.83
	Full	6	0.99	728.02	743.65	716.02	0.94	Trial type	0.81	1.32	0.62	0.54
								Age * Trial type	−0.14	1.86	−0.07	0.94
Case G	Restricted	5	0.74	1,122.6	1,135.7	1,112.6		Age	38.37	2.02	18.97	**<0.001**
	Full	6	0.74	1,124.2	1,139.8	1,112.2	0.48	Trial type	87.7	1.38	63.77	**<0.001**
								Age * Trial type	71.43	2.3	31.03	**<0.001**
Case H	Restricted	5	0.97	886.97	900.00	876.97		Age	99.55	1.87	53.33	**<0.001**
	Full	6	0.99	775.52	791.16	763.52	**113.45**	Trial type	100.38	1.33	75.73	**<0.001**
								Age * Trial type	114.68	2.21	51.98	**<0.001**

*Full = full model including interaction term. Restricted = restricted model without the interaction term. For R^2^, the marginal coefficient is presented because it expresses how much variance is explained by the fixed factors for generalized linear mixed models with random slopes (Johnson, 2014). AIC = Akaike information criterion. BIC = Bayesian information criterion. Change in χ^2^ (Δχ^2^) was calculated relative to the full model. Boldface type indicates p < .05. Trial-level multilevel model fixed effects on the right.*

Thus, the question arises: How can we remedy this in the visual presentation of the Brinley plot? Specifically, there are two problems with the Brinley plot that make it difficult to interpret from the standpoint of identifying study-level effects. First, data that are coupled at the study level (congruent and incongruent trials) are often decoupled when displayed in Brinley plots (see for example Rey-Mermet et al., 2018). Because the Age by Trial Type interaction relies on a comparison of congruent to incongruent trials, at the level of the study, eliminating the study-level connection between congruent and incongruent trials also eliminates clear evidence of the interaction from the plot. When the study conditions are connected (e.g., in Verhaeghen, 2011), they are often difficult to visually interpret because of the condition trendlines. Second, it is difficult to gauge the nature of age differences in the plot (i.e., either overall or task-specific) without a clear reference line which, again, is not always present in Brinley plots. Modifications to the Brinley plot can remedy these problems.

In the modified Brinley plot (on the right in Figure 2), the addition of study-level slopes connecting congruent and incongruent trial means and a no-age-difference reference line allow for a clearer presentation of the effects present in the data and as supported by the multilevel model. With the modified Brinley plot, it is evident that the individual studies on the whole fall above the reference line, indicating age differences across studies. The slower response times for the incongruent trials compared to the congruent trials are also now obviously present for all of the studies; in the traditional (uncoupled) Brinley plot, congruent and incongruent means are interspersed with no clear way of knowing if a consistent Stroop effect has been found across studies. Finally, and perhaps most importantly, the study-specific slopes connecting congruent and incongruent means are steeper than the diagonal reference line, indicating that the critical interactions are present at the study level. Thus, in contrast to the standard Brinley plot, which would convey that there are no age differences in the Stroop effect present, the modified Brinley plot clearly reflects the underlying patterns of the data which are supported by the multilevel model results.

Ultimately, to foreshadow our findings, the standard Brinley plot is not well suited to identify or display the underlying data structure when there are within group manipulations. Study-level interactions are challenging to identify because conditions are decoupled and group effects are not apparent in the absence of a reference line. These issues, at least visually, are solved by using a modified Brinley plot that includes study-specific slopes that connect conditions from the same studies and a no-group-difference diagonal identity line for reference. Multilevel models will find all of the relevant effects, especially if applied to trial-level data, and have the advantage of incorporating study-level moderators that might account for slope heterogeneity.

## Materials and Methods

### Simulated Data

The data were simulated using a younger adult response time mean of 700 ms for the congruent trials and 800 ms for the incongruent trials with standard deviations of 150 ms and 150 ms, respectively. For the older adult participants, data were simulated with a response time mean of 750 ms and 900 ms for congruent and incongruent trials, respectively (standard deviations set to 200 ms and 200 ms, respectively).^2^ Intertrial correlations were assumed to have an autoregressive (*r* = 0.60) structure. Each trial type was generated to have 20 trials (all assumed to be correct). Between-study variability was controlled using a “disturbance” parameter, which was set independently for each Age by Trial Type combination. All data and analyses are made available on OSF (see text foot note 5).

For each multi-study case, presented below, 50 studies were generated; all studies had the same underlying means, standard deviations, number of trials, and autocorrelation unless otherwise specified. All sample sizes per study were the same, 50 younger (faster) and 50 older (slower) participants. The parameters (Age by Trial Type combination means and standard deviations) of the simulated data cases are presented in Table 1.

### General Analysis Procedure

As noted, in Brinley analyses (e.g., Cerella, 1994; Cerella and Hale, 1994), the average performance of younger and older adults are plotted against each other for each study and hierarchical modeling is used to determine whether the data can be reliably fitted with a single line or two different lines. If two lines are necessary (i.e., one for interference trials and one for baseline trials), it suggests an age-related effect above and beyond general age effects on processing speed. In contrast, if a single line is sufficient, this suggests a lack of an age difference in the Stroop effect. To statistically determine whether one or two regression lines were necessary to explain the data in each simulated case, we used hierarchical modeling (as in Verhaeghen, 2011; Rey-Mermet and Gade, 2018). To account for within-study and between-study variances, multilevel regression models with random intercepts and slopes were computed using the lme4 package in R (Bates et al., 2015) and the following equation:


$$RT_{o\,l\,d\,e\,r,i\,j}=\beta_{0}+\beta_{1}TrialType+\beta_{2}RT_{y\,o\,u\,n\,g,i\,j}+\beta_{3}TrialTypeRT_{y\,o\,u\,n\,g,i\,j}+\left(b_{0\,i}+b_{1\,i}RT_{y\,o\,u\,n\,g,i\,j}+\in_{i\,j}\right)$$

where *RT*_*older*,*ij*_ is the average response time of the older adult group from the condition *j* in study *i*, *RT*_*young*,*ij*_ is the average response time of the younger adult group from the condition *j* in study *i*, Trial Type is the variable representing congruent and incongruent trials, β_0_ is the intercept, β_1_ is the effect of Trial Type (congruent vs. incongruent) on the intercept, β_2_ is the slope relating the older adult group to the younger adult group, β_3_ is the effect of Trial Type on the slope,*b*_*0i*_ is the random intercept for study *i*,*b*_*1i*_is the random slope for study *i*, and ∈_*ij*_ is the residual for the condition *j* in study *i*. We compared the full model against a restricted model in which the interaction term was removed (see Rey-Mermet and Gade, 2018). Model fit and comparisons were evaluated based on the *R*^2^ and Δχ^2^-tests on the nested models. If the more complex model (the full model, including the interaction term) yielded a reduction in χ^2^ that was significant given the loss of degrees of freedom, then it was accepted as having a better fit, i.e., two lines are necessary to account for the data, and indicate the presence of an age-related increase in Stroop effects. If the Δχ^2^-test was not significant, this suggested that the restricted model without the interaction term had a better fit than the full model, i.e., that one line would be sufficient to account for the data, and hence indicated the absence of a disproportionate age difference in the Stroop effect.

Importantly, as noted earlier, we also used multilevel modeling of simulated trial-level response times for comparison to the model comparison analyses described above. Models were conducted using the lme package (Pinheiro et al., 2021) in R. Participant and dataset were allowed to vary randomly and Age (Younger, Older) and Trial Type (Congruent, Incongruent) were included as fixed effect factors [RT ∼ 1 + Age + Trial Type + Age*Trial Type + (1 | Participant) + (1 | Dataset)]. The equation was as follows:Level 1 (Trial):


$$R\,T_{i\,j\,k}=\pi_{0\,j\,k}+\pi_{1\,j\,k}\,T\,r\,i\,a\,l\,T\,y\,p\,e_{i\,j\,k}+e_{i\,j\,k}$$

Level 2 (Participant):


$$\pi_{0\,j\,k}=\,\beta_{00\,k}+\beta_{01\,k}\,A\,g\,e_{j\,k}+r_{0\,j\,k}$$

$$\pi_{1\,j\,k}=\,\beta_{10\,k}+\beta_{11\,k}\,A\,g\,e_{j\,k}$$

Level 3 (Dataset):


$$\beta_{00\,k}=\,\gamma_{000}+u_{00\,k}$$

$$\beta_{01\,k}=\,\gamma_{010}$$

$$\beta_{10\,k}=\,\gamma_{100}$$

$$\beta_{11\,k}=\,\gamma_{110}$$

where *RT*_*ijk*_ is the response time of trial *i* for participant *j* in dataset *k*. *TrialType*_*ijk*_ is the variable representing the trial type (congruent or incongruent) for trial *i* for participant *j* in dataset *k*. *Age*_*jk*_ is the variable representing the age (younger or older) of participant *j* in dataset *k*. Including participant as a random effect in the model accounts for overall processing speed, as each participant’s mean response time is controlled for. The main question was whether this model would yield the same results on the same data, with predetermined effects, as the Brinley analyses (i.e., model comparisons) described above.^3^

## Results

### Case A

Case A represented a multi-study simulation of the underlying data structure described above such that both main effects of Age and Trial Type, and their interaction were present. All studies had the same population interaction underlying their samples. The studies differed only randomly such that the effect on the data for this case was to increase or decrease all of the means for a given study by a constant and randomly determined amount (a random shift in the grand mean across studies).^4^ This would be akin to differences across studies in the general difficulty of the task, which could occur for a variety of reasons (differences in the task parameters, conditions of testing, samples, etc.). The important issue addressed here is whether the Brinley plot for the study means would reveal the Age by Trial Type interaction that underlies the data.

**FIGURE 3:**
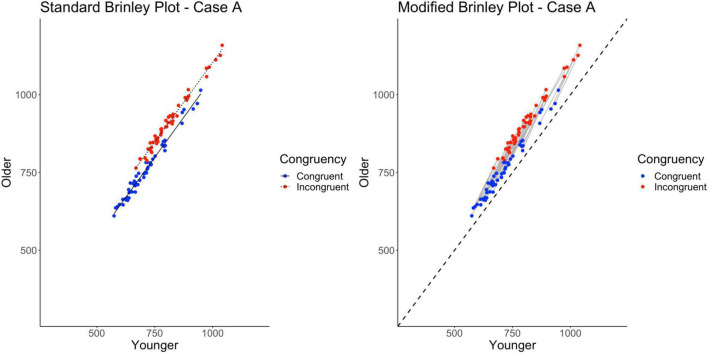
Standard and modified Brinley plots—Case A data. Standard and modified Brinley plots (left and right, respectively) for the Case A simulated data which was simulated such that all studies had the same interaction. Means (SD) used for Case A were as follows: Young Congruent = 700 (150), Young Incongruent = 800 (150), Old Congruent = 750 (200), Old Incongruent = 900 (200).

As shown on the left in Figure 3 and Table 2, despite the fact that the data were specifically generated with an Age by Trial Type interaction present, it does not appear in the plot, at least not as expected (non-parallel slopes). Furthermore, the Brinley-based analyses showed a better model fit for the restricted model as compared to the full model. Model fit and comparisons were evaluated based on the Δχ^2^-test on the nested models (along with *R*^2^, AIC, BIC, and deviance values). The Δχ^2^− test did not reach significance, *p* = 0.97, indicating that the restricted model without the interaction term had a better fit than the full model, i.e., that one line would be sufficient to account for the data, and hence indicates the absence of disproportionate group differences in the Stroop effect, above and beyond general slowing. Thus, these results replicate prior findings using Brinley analyses to examine age differences in attentional control and suggest that there is no evidence for group differences in the Stroop effect in older adults, as all data were well captured with a single regression line (Verhaeghen and De Meersman, 1998; Verhaeghen, 2011; Rey-Mermet and Gade, 2018).

As mentioned, one issue with the Brinley plot is that it treats trial types as if they were independent. This is true even when conditions from the same study are connected, as the analyses do not factor in the study condition covariance. As within-person (and within-study) variables, they are clearly not independent. If these variables are treated correctly, as in a multilevel model of the data, the interaction (which is built into the simulated data) should emerge. Furthermore, if trial-level data are available, the data can be examined using a three-level model: trials nested within participants and participants nested within studies. Indeed, such a model takes into account the entire dependence structure in the data and confirms the presence of the interaction in Case A, as simulated (see right side of Table 2).

As noted earlier, we created a modified Brinley plot which connects trial types from each study and includes a no-group-differences reference line (i.e., a diagonal identity line). Studies that fall above this line show the main effect of Age, and studies that show a steeper slope than the reference line are those with the critical Age by Trial Type interaction. This is precisely what is shown in the modified Brinley plot (see right side of Figure 3).

### Case B

Case B explored an implication of Case A, specifically that increasing the magnitude of the Age by Trial Type interaction in the individual studies would still yield a standard Brinley plot with parallel group slopes. Instead, the study-level directed lines would have greater slopes relative to the reference line. The only change made from Case A was that the difference between trial types was exaggerated for the older adult, or slower group, sample so as to exaggerate the within-study interaction effect (as compared to Case A; see Table 1).

**FIGURE 4:**
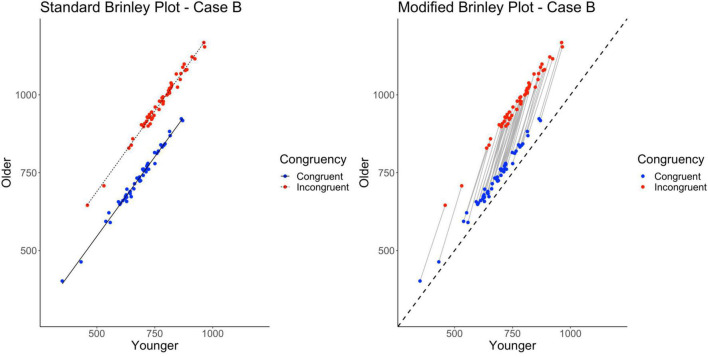
Standard and modified Brinley plots—Case B data. Standard and modified Brinley plots (left and right, respectively) for the Case B simulated data which was simulated such that the magnitude of the interaction was increased. Means (SD) used for Case B were as follows: Young Congruent = 700 (150), Young Incongruent = 800 (150), Old Congruent = 750 (200), Old Incongruent = 1,000 (200).

Once again, as shown on the left in Figure 4 and Table 2, despite the fact that the Age by Trial Type interaction is generated (and exaggerated) in the Case B data, it does not appear in the standard Brinley plot in the form of non-parallel slopes. Instead, the two slope lines are displaced vertically to a greater degree than was true for Case A. Like Case A, however, the model comparisons yield a non-significant Δχ^2^-test, *p* = 0.91, indicating that the full model with the interaction term did not have a better fit than the restricted model, i.e., that one line would be sufficient to account for the data.

As with the Case A data, the results of the trial-level multilevel model and the modified Brinley plot do not converge with these results. As shown on the right in Table 2 and Figure 4, there is a robust interaction term and the study-level lines run clearly non-parallel to the reference line.

Ultimately, Case B indicates that even in a situation where the data were generated with an exaggerated interaction for all studies, the results of the Brinley plot and model comparison analyses fail to converge with the trial-level multilevel model and modified Brinley plot.

### Case C

Case C explores a second implication of Case A (and Case B) that if the interaction was absent in the individual studies, then the vertical separation of the slope lines in a standard Brinley plot should disappear, and, all individual-study slopes in the modified Brinley plot should be similar to the reference line. In Case C, the underlying means had main effects of Age and Trial Type but the difference between trial types was the same across participant groups (i.e., no interaction; see Table 1).

**FIGURE 5:**
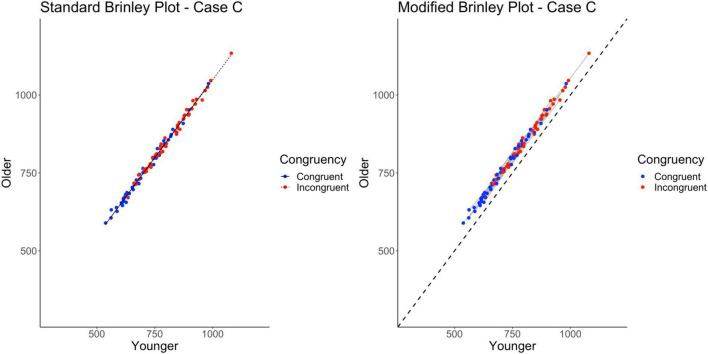
Standard and modified Brinley plots—Case C data. Standard and modified Brinley plots (left and right, respectively) for the Case C simulated data which was simulated such that there was no interaction present. Means (SD) used for Case C were as follows: Young Congruent = 700 (150), Young Incongruent = 800 (150), Old Congruent = 750 (200), Old Incongruent = 850 (200).

As shown on the left in Figure 5, and as expected, all of the data fall along a single line. The longer incongruent trial response times relative to congruent trial response times (horizontal displacement of the incongruent trials) is evidence of the overall Stroop effect. The overall Age effect is more difficult to see without the diagonal no-age-difference reference line (which the modified Brinley plot provides). As shown on the left in Table 2, the model comparisons yield a non-significant Δχ^2^-test, *p* = 0.18, indicating that the restricted model without the interaction term had a better fit than the full model, i.e., that one line would be sufficient to account for the data. Although the standard Brinley plot is still lacking in providing the viewer with a complete understanding of the underlying data structure, the results of the model comparison analysis do provide an appropriate conclusion in this case.

The results of the trial-level multilevel model provide converging evidence, as shown in Table 2, such that the two main effects are significant but not the interaction. The modified Brinley plot for these data, shown on the right in Figure 5, now shows all of the individual study slopes falling along the general slope line that is similar to the reference line. The overall age effect is also clear because all study-specific lines fall above the reference line.

### Case D

Case D represents a multi-study simulation of data with an underlying structure with only a main effect of Trial Type in the data; there were no Age effects or interactions.

**FIGURE 6:**
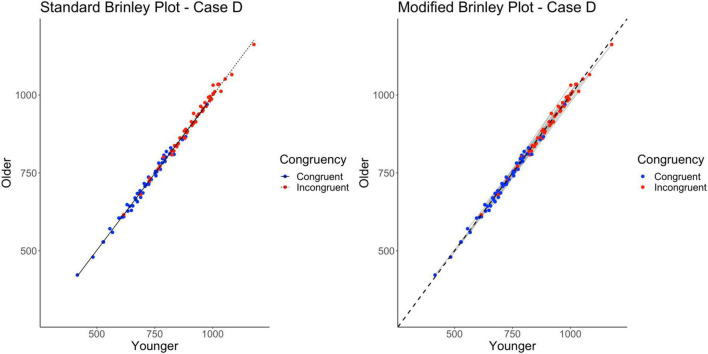
Standard and modified Brinley plots—Case D data. Standard and modified Brinley plots (left and right, respectively) for the Case D simulated data which was simulated such that there was only a main effect of trial type present. Means (SD) used for Case D were as follows: Young Congruent = 700 (150), Young Incongruent = 900 (150), Old Congruent = 700 (150), Old Incongruent = 900 (150).

Without a reference line, however, the Brinley plot for Case D (shown on left in Figure 6) appears very similar to the standard Brinley plot for Case C. Again, the horizontal displacement of the incongruent trials is evidence of the overall effect of Trial Type and the overall Age effect (or lack thereof) is difficult to see without a reference line. As shown on the left in Table 2, The model comparisons similarly yield a non-significant Δχ^2^-test, *p* = 0.71, indicating that the restricted model without the interaction term had a better fit than the full model, again, an appropriate conclusion in this case.

The results of the trial-level multilevel model, as shown on the right in Table 2, show a significant effect of Trial Type but neither the main effect of Age nor the Age by Trial Type interaction reached significance. The absence of an Age effect is now clearly identified in the modified Brinley plot, shown on the right in Figure 6, by the lack of difference between the study-level lines and the reference line. The absence of the interaction is evident by the study-level slopes being similar to the reference line slope.

### Case E

Case E represents a multi-study simulation of data with only a main effect of Age present in the data (i.e., no effect of Trial Type and no interaction).

**FIGURE 7:**
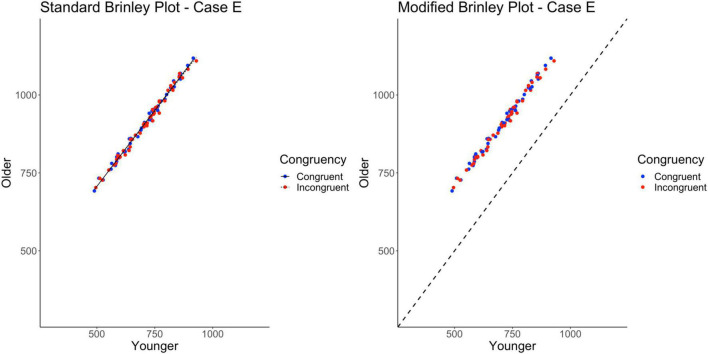
Standard and modified Brinley plots—Case E data. Standard and modified Brinley plots (left and right, respectively) for the Case E simulated data which was simulated such that there was only a main effect of age. Means (SD) used for Case E were as follows: Young Congruent = 700 (150), Young Incongruent = 700 (150), Old Congruent = 900 (150), Old Incongruent = 900 (150).

The standard Brinley plot, shown on the left in Figure 7, shows that the slope of the lines are nearly identical, as has been true so far for all of the cases. The lack of horizontal displacement of the incongruent trial means relative to the congruent trial means indicates the lack of a Trial Type effect. However, it is again difficult to discern the Age effect in the absence of a reference line. As shown on the left in Table 2, the model comparisons similarly yield a non-significant Δχ^2^-test, *p* = 0.43, indicating that the restricted model without the interaction term had a better fit than the full model, another appropriate statistical conclusion given the simulated properties of the data.

The results of the trial-level multilevel model, as shown in Table 2, confirm that the main effect of Age is the only significant effect in the data. The presence of the Age effect is easy to see in the modified Brinley plot, as shown on the right in Figure 7, because all study-level data are above the reference line. Furthermore, the study-level lines are extremely short, indicating the absence of any Stroop effects.

### Case F

Case F represents a multi-study simulation of data with only null effects at the study-level (i.e., underlying means identical for all groups).

**FIGURE 8:**
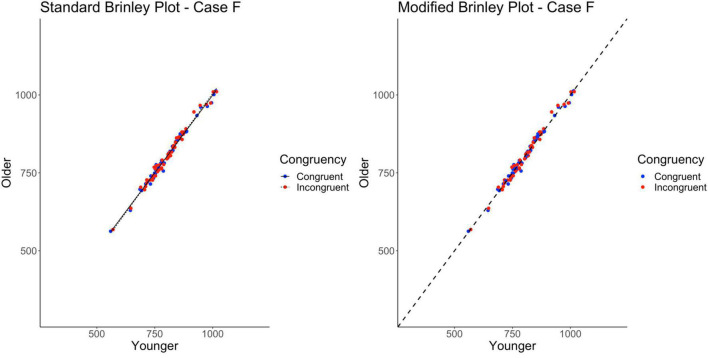
Standard and modified Brinley plots—Case F data. Standard and modified Brinley plots (left and right, respectively) for the Case F simulated data which was simulated such that all effects were null. Means (SD) used for Case F were as follows: Young Congruent = 800 (150), Young Incongruent = 800 (150), Old Congruent = 800 (150), Old Incongruent = 800 (150).

The standard Brinley plot, shown on the left in Figure 8, shows no horizontal displacement of the incongruent trials indicating no Stroop effects in the data. The absence of an Age effect is not easy to see without a reference line and the absence of an interaction is not easy to see without the study-level slopes. As shown on the left in Table 2, the model comparisons similarly yield a non-significant Δχ^2^test, *p* = 0.49, correctly indicating that the restricted model without the interaction term had a better fit than the full model, i.e., that one line would be sufficient to account for the data. Importantly, however, both the full and restricted Brinley models show a significant β_2_*RT*_*young*,*ij*_, *p* < 0.001, an effect which was not present in the underlying data structure.

In contrast, the results of the trial-level multilevel model, as shown on the right in Table 2, confirms the lack of any significant effects in the data. The modified Brinley plot, as shown on the right in Figure 8, clearly displays the absence of an Age effect and the absence of a Trial Type effect.

### Case G

As mentioned above, Cases A-F induced between-study variability by adding the same random constant to all means within a study. Although useful, this may not be particularly realistic. It could be the case that studies are different in this general way but also different at the individual means level. Case G explores the possibility that studies differ at the level of the individual means as well. In this case, two sources of between-study variability are simulated: (a) the general random study differences that have been present in the other cases and (b) random differences that are specific to the Age by Trial Type interaction. This will have the effect of inducing more variability in the means. The underlying effects, however, are identical to Case A (i.e., both main effects and the interaction were present in the data).

**FIGURE 9:**
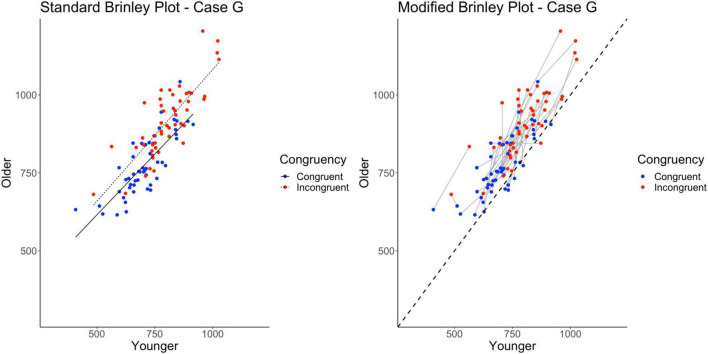
Standard and modified Brinley plots—Case G data. Standard and modified Brinley plots (left and right, respectively) for the Case G simulated data which was simulated such that study interaction magnitudes differed randomly. Means (SD) used for Case G were as follows: Young Congruent = 700 (150), Young Incongruent = 800 (150), Old Congruent = 750 (200), Old Incongruent = 900 (200).

The standard Brinley plot, shown on left in Figure 9, shows more variability around the slope lines for the congruent and incongruent means. Other than that, however, the Brinley plot for these data is not any more interpretable than Brinley plots for the other cases. The slopes are still parallel and it is impossible to tell if study-level interactions are present. As shown on the left in Table 2, the model comparisons similarly yield a non-significant Δχ^2^-test, *p* = 0.93, indicating that the restricted model without the interaction term had a better fit than the full model, i.e., that one line would be sufficient to account for the data. Importantly, however, the full Brinley model does not show a significant effect of Trial Type (see Table 3), an effect which was present in the underlying data structure.

**TABLE 3:** Model comparison analysis fixed effect estimates.

Data and analysis	β_0_	β_1_	β_2_	β_3_
**Case A**				
Restricted	**55.60 (11.45)**	**50.03 (2.21)**	**0.99 (0.02)**	–
Full	**66.43 (12.77)**	**26.80 (12.56)**	**0.98 (0.02)**	0.03 (0.02)
**Case B**				
Restricted	**76.47 (12.75)**	**153.61 (2.46)**	**0.96 (0.02)**	–
Full	**60.82 (14.22)**	**185.69 (12.70)**	**0.99 (0.02)**	**0.04 (0.02)**
**Case C**				
Restricted	**69.28 (12.21)**	4.01 (2.23)	**0.98 (0.02)**	–
Full	**71.17 (13.27)**	−0.28 (11.43)	**0.97 (0.02)**	0.01 (0.01)
**Case D**				
Restricted	15.66 (8.16)	0.24 (2.77)	**0.98 (0.01**)	–
Full	15.42 (9.65)	0.65 (10.03)	**0.98 (0.01)**	−0.001 (0.01)
**Case E**				
Restricted	**188.42 (8.08)**	2.37 (1.31)	**1.01 (0.01)**	–
Full	**183.55 (9.37)**	12.09 (9.56)	**1.02 (0.01)**	−0.01 (0.01)
**Case F**				
Restricted	−3.30 (11.21)	0.21 (1.37)	**1.00 (0.01)**	–
Full	−11.15 (12.51)	15.98 (11.18)	**1.01 (0.02)**	−0.02 (0.01)
**Case G**				
Restricted	**225.76 (45.53)**	**81.65 (13.12)**	**0.72 (0.07)**	–
Full	**174.45 (57.57)**	197.61 (79.46)	**0.80 (0.08)**	−0.16 (0.11)
**Case H**				
Restricted	**−89.92 (24.86)**	**85.80 (4.86)**	**1.24 (0.03)**	–
Full	**96.02 (24.27)**	**−292.04 (26.90)**	**1.00 (0.03)**	**0.46 (0.03)**

*Restricted vs. full model comparison fixed effects. Standard errors presented in parentheses. β_0_ is the intercept, β_1_ is the effect of trial type (interference vs. baseline) on the intercept, β_2_ is the slope relating older to young adults, β_3_ is the effect of trial type for the Brinley analysis on the slope. Boldface type indicates p < 0.05.*

In contrast, the results of the trial-level multilevel model, as shown on the right in Table 2, confirms the presence of the Age by Trial Type interaction in part because it models all sources of variability in the data. It is attenuated as compared to Case A because of the greater variability (all random) induced in this set of simulated data but is nevertheless present. The additional variability imposed on the data induces heterogeneity in the study-level lines. Still, one can see that their aggregate slope is greater than the reference line in the modified Brinley plot, as shown on the right in Figure 9. The main effects of Age and Trial Type are likewise easy to see in the plot by the location of the means relative to the reference line and the horizontal displacement of the congruent and incongruent means.

### Case H

Cases A-G had parallel slopes for the congruent and incongruent trials in the standard Brinley plot. However, some Brinley plots in prior research have shown non-parallel lines for the trial type means (e.g., the “divided attention” tasks in Verhaeghen, 2011 and Stop-signal and Go/No-Go tasks in Rey-Mermet and Gade, 2018). One goal of Case H was to further examine when this may occur. In the previous cases, non-parallelism does not occur, even when interactions are present in the individual studies, because the studies differ randomly in their means (either in the grand means or the condition-specific means). If the magnitude of the interaction varies across studies in a systematic way, however, this should produce non-parallel lines in a standard Brinley plot. This might happen, for example, if studies varied in the kinds of Stroop tasks they used so that some are more sensitive than others to Age effects, as might be expected in the empirical literature. The underlying means in Case H have the interaction pattern from Case A (along with the two main effects), but differences are imposed at the study level to make the interaction stronger in some studies than others.

**FIGURE 10:**
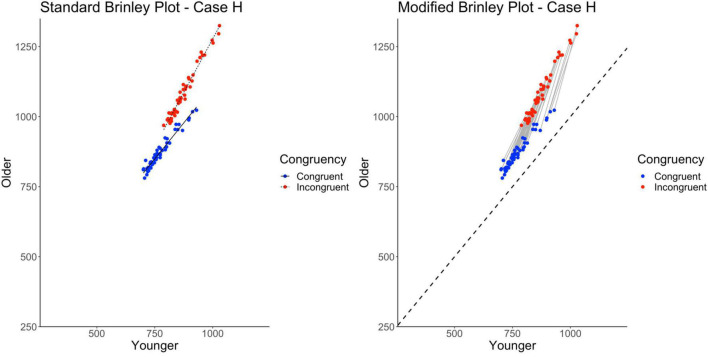
Standard and modified Brinley plots—Case H data. Standard and modified Brinley plots (left and right, respectively) for the Case H simulated data which was simulated such that stronger interactions were systematically introduced for some studies but not others. Means (SD) used for Case G were as follows: Young Congruent = 700 (150), Young Incongruent = 800 (150), Old Congruent = 750 (200), Old Incongruent = 900 (200).

Finally, the standard Brinley plot, shown on the left in Figure 10, shows Trial Type slope lines diverging from parallel. However, it is important to note that this has been induced by the between-study differences imposed on the Case H data. As shown on the left in Table 2, the model comparisons similarly yield a significant Δχ^2^-test, *p* < 0.001, indicating that the full model with the interaction term at last had a better fit than the restricted model, i.e., that two lines would be necessary to account for the data.

The results of the trial-level multilevel model, shown on the right in Table 2, converge with these results and confirm the presence of the Age by Trial Type interaction. Furthermore, if there were known task differences across studies that might explain the varying interaction effects, they could be incorporated into the model. Now, in the modified Brinley plot, as shown on the right in Figure 10, the individual study slopes are clearly different from the reference slope (presence of study-level interactions) but also varying in magnitude in a systematic way across studies (increasing slope as congruent response time increases).

## Discussion

The present study extends upon the work of Perfect (1994) and Nicosia et al. (2021) by examining the circumstances necessary to produce converging results indicating a larger Stroop effect for older adults than younger adults (an Age by Trial Type interaction) using Brinley analyses and multilevel models. Across eight cases of systematically simulated data, four of which were generated with an interaction present, the Brinley plot and accompanying analyses failed in all cases but one to capture the underlying data structure and produce converging results with the trial-level multilevel models. The only case in which the Brinley plots and model comparison analyses yielded results consistent with the underlying structure of the generated data and the trial-level multilevel models was in Case H when all studies were simulated with the Age by Trial Type interaction, and the corresponding main effects, and differences were imposed at the study level to make that interaction stronger in some studies than others. Thus, it appears that the sole circumstance in our simulations under which Brinley plots and the accompanying model comparison analyses are able to detect an Age by Trial Type interaction is when there is systematic variation in the magnitude of the interaction across studies. Given that the Brinley-type analyses did not yield results consistent with the underlying data structure or trial-level multilevel models for Cases A, B, G, or the meta-analysis data, it is clear that Brinley plots and their accompanying analyses should be used with caution by cognitive aging researchers.

As mentioned earlier, the Stroop task was taken as an example here but this issue persists across many different tasks that assess attentional control. For example, the Simon task, Flanker task, and Switching tasks all tap into various components of attentional control and have been subjected to Brinley plot meta-analyses (e.g., Verhaeghen and De Meersman, 1998; Verhaeghen, 2011; Rey-Mermet and Gade, 2018). These meta-analyses, like those for the Stroop task, have largely attributed apparent age differences to processing speed differences, and have dismissed a potential role for specific age-related mechanisms. Herein we have used the Stroop task as an example because it has been subject to controversy in the literature despite being dubbed the quintessential task for investigating attentional control (see MacLeod, 1991). Nevertheless, one should be cautious not to assume that all attentional control tasks tap into the same mechanism when approaching how individuals select relevant information and resist distracting information, and as a function of age.

### What Can Brinley Plots Tell Us About Cognitive Aging?

Based on the results of the present study, it appears that Brinley plots, and their accompanying analyses, are useful for telling us when variability across studies exists in the magnitude of the interference effect produced across age groups. Despite claims in the literature, Brinley plots do not, however, tell us when a disproportionate group difference in an interference effect (i.e., an interaction) is present. As shown, the only case when Brinley plots visually indicate, and substantiate with the restricted vs. full model comparison analysis, that two lines are necessary to account for the data was in Case H. Although Case H used the same underlying data structure as Case A (and, by extension, Cases B and G), in which the data were generated with an interaction present, Brinley analyses did not indicate support for two regression lines. The critical difference between Cases A, B, and G (where the data were generated with interactions present yet Brinley analyses did not yield this result) and Case H (where the data were generated with interactions present and the Brinley analysis *did* show this result) was that the magnitude of the interaction varied *across studies* in Case H. This is consistent with the result of Perfect (1994) such that Brinley plots only reflect study-level interactions when the effect is *extremely* exaggerated; we extend upon these results because our simulated data show that the variability in the interaction term across studies included seems to drive when the restricted or full model should be accepted.

Moreover, the restricted vs. full model comparisons, which commonly accompany Brinley plots, are also problematic. As Fisk et al. (1992) and Perfect (1994) have already pointed out: “because of range effects, the amount of variance accounted for can be misleading, and ceiling effects in amount of variance explained can mask the psychological reality of the aging process. If a single function can account for around 95% of the variance, then more complex models can appear redundant because at best they only add another 5% of the variance.” And, because these types of models use younger adult response times to predict older adult response times (rather than using an age group factor to predict response times), large correlations and ceiling effects in the amount of variance left to be accounted for will always be present. Additionally, not only did Brinley plots and the accompanying model comparison analyses *not* show interactions when they were present in the data, there were also two cases where the model comparison analyses misrepresented the data. First, in Cases D and F, when only a Trial Type effect and null effects were present in the simulated data, respectively, both the restricted and full models yielded significant effects of Age whereas the trial-level multilevel model did not misrepresent the data in this way. Second, in Case G, when both main effects and the interaction were present in the simulated data, the full model did not show a significant effect of Trial Type, an effect which was present in the underlying data structure and detected using the trial-level multilevel model. Therefore, it is quite clear that, aside from the problems with the Brinley plots, that even the seemingly objective analyses which accompany them (i.e., restricted vs. full model comparisons) are flawed and mislead researchers as to the effects present in their data.

It should also be noted that although we have simulated these data as younger adults vs. older adults, this work is also relevant to any situation in which speed of processing is confounded with group status; faster vs. slower populations, and, for example individuals with ADHD or schizophrenia as compared to neurotypical controls.

### The Modified Brinley Plot

Ultimately, as shown here, the standard Brinley plot is not well suited to identify or display the underlying data structure. Study-level interactions are impossible to identify because conditions are decoupled and Age effects are difficult to identify in the absence of a reference or identity line. Both problems are solved by using a modified Brinley plot that includes study-specific slopes linking trial types and a no-age-difference reference. Multilevel models will find all of the relevant effects, especially if applied to trial-level data, and have the advantage of incorporating study-level moderators that might account for slope heterogeneity.

### Recommendations to Researchers

Although we considered eight different cases of simulated data, along with meta-analysis data, with varying underlying structures, we encourage other researchers to access the code and data available on OSF (see text foot note 1) and explore further issues of interest. Indeed, only a finite number of data cases were explored here such that questions regarding accuracy and potential speed-accuracy tradeoff effects could be addressed in future work (see Ratcliff and McKoon, 2000; Horn et al., 2013 for further discussion). Otherwise, we advise researchers who study cognitive aging, or any type of data where group differences in processing speed present an issue, to avoid the use of Brinley plots and their accompanying model comparison analyses unless aiming to assess whether the magnitude of the interaction varies across studies. Additionally, in these rare cases where one might employ the use of Brinley-type analyses, we encourage researchers to additionally (1) present the data with study-specific slopes linking conditions within a study and include a no-age-difference reference so as to make the underlying structure of the data more apparent to readers and (2) run multilevel models, with trial-level data if possible, to best reflect the effects present in the data.

## Conclusion

Cognitive aging researchers have been challenged with demonstrating effects above and beyond global slowing since before this issue was brought to the forefront of gerontological research by Cerella (1990). Indeed, this has proved to be a difficult task as many decades of research have unveiled. Nevertheless, Brinley plots do not appear to be the solution: data that have been generated specifically with an underlying interaction term fail to produce a plot which requires two functions to fit the data and, because of a combination of range effects and the “unprincipled fitting of global parameter values” (Perfect, 1994), Brinley plots and the parameter estimates generated in this fashion paint a distorted picture of the underlying data and the true effects present.

As put so eloquently by Perfect (1994) several decades ago: “Consideration of the literature shows that Brinley plots have been conducted in ways that would lead to a single factor model being accepted irrespective of whether such a model is appropriate. Measurement error, plots with few points, tasks that are sampled over different ranges of response latency, and the fitting of several equations with more than one parameter all lead to the premature acceptance of global models of cognitive change with age. The siren call of correlations of 0.95 and above should be resisted. Like the mermaid’s song, they are an alluring yet dangerous diversion.”

## Data Availability Statement

The original contributions presented in the study are included in the article/supplementary material, further inquiries can be directed to the corresponding author/s.

## Author Contributions

JN analyzed the data and wrote the manuscript. EC-S assisted in writing the manuscript. MS assisted with conceptualization and data analysis. All authors contributed to the article and approved the submitted version.

## Conflict of Interest

The authors declare that the research was conducted in the absence of any commercial or financial relationships that could be construed as a potential conflict of interest.

## Publisher’s Note

All claims expressed in this article are solely those of the authors and do not necessarily represent those of their affiliated organizations, or those of the publisher, the editors and the reviewers. Any product that may be evaluated in this article, or claim that may be made by its manufacturer, is not guaranteed or endorsed by the publisher.

## Footnotes

1 An alternative approach to controlling for overall speed differences between groups is to use standardized (z-scored) response times, which rescales each trial’s response time to the mean and standard deviation for that individual. This puts all participants, across age groups, on a common scale. Therefore, analyses of standardized response times eliminate general speed differences and allow the researcher to examine any age interactions (e.g., with task or condition) above and beyond slowing. See Faust et al. (1999) for further information.

2 These values were selected because they closely approximate the means and standard deviations reported in the color-naming Stroop task literature (see Nicosia et al., 2021). However, we encourage readers to access the code on OSF and use different values as well.

3 In the models presented here, we restrict the random model to intercepts. However, more complex random models are possible and would allow for a deeper exploration of possible moderators.

4 A “disturbance” term was used to induce between-study variability. The disturbance term was a value selected from a randomly generated normal distribution. For each study, the disturbance term was added to the means to randomly shift the grand mean across studies. For additional information, please see code posted on OSF: https://osf.io/zxus8/
